# JrPHL8-JrWRKY4-JrSTH2L module regulates resistance to *Colletotrichum gloeosporioides* in walnut

**DOI:** 10.1093/hr/uhae148

**Published:** 2024-05-28

**Authors:** Yutian Mu, Yuhui Dong, Xichen Li, Andi Gong, Haiyi Yu, Changxi Wang, Jianning Liu, Qiang Liang, Keqiang Yang, Hongcheng Fang

**Affiliations:** College of Forestry, Shandong Agricultural University, Taian 271018, Shandong, China; College of Forestry, Shandong Agricultural University, Taian 271018, Shandong, China; Mountain Tai Forest Ecosystem Research Station of State Forestry and Grassland Administration, Shandong Agricultural University, Taian 271018, Shandong, China; State Forestry and Grassland Administration Key Laboratory of Silviculture in Downstream Areas of the Yellow River, Taian 271018, Shandong, China; College of Forestry, Shandong Agricultural University, Taian 271018, Shandong, China; College of Forestry, Shandong Agricultural University, Taian 271018, Shandong, China; College of Forestry, Shandong Agricultural University, Taian 271018, Shandong, China; College of Forestry, Shandong Agricultural University, Taian 271018, Shandong, China; College of Forestry, Shandong Agricultural University, Taian 271018, Shandong, China; College of Forestry, Shandong Agricultural University, Taian 271018, Shandong, China; Mountain Tai Forest Ecosystem Research Station of State Forestry and Grassland Administration, Shandong Agricultural University, Taian 271018, Shandong, China; State Forestry and Grassland Administration Key Laboratory of Silviculture in Downstream Areas of the Yellow River, Taian 271018, Shandong, China; College of Forestry, Shandong Agricultural University, Taian 271018, Shandong, China; Mountain Tai Forest Ecosystem Research Station of State Forestry and Grassland Administration, Shandong Agricultural University, Taian 271018, Shandong, China; State Forestry and Grassland Administration Key Laboratory of Silviculture in Downstream Areas of the Yellow River, Taian 271018, Shandong, China; College of Forestry, Shandong Agricultural University, Taian 271018, Shandong, China; Mountain Tai Forest Ecosystem Research Station of State Forestry and Grassland Administration, Shandong Agricultural University, Taian 271018, Shandong, China; State Forestry and Grassland Administration Key Laboratory of Silviculture in Downstream Areas of the Yellow River, Taian 271018, Shandong, China

## Abstract

Walnut anthracnose (*Colletotrichum gloeosporioides*) reduces walnut yield and quality and seriously threatens the healthy development of the walnut industry. WRKY transcription factors (TFs) are crucial regulatory factors involved in plant-pathogen interactions. Our previous transcriptome analysis results indicate that JrWRKY4 responds to infection by *C. gloeosporioides*, but its specific regulatory network and disease resistance mechanism are still unclear. Herein, the characteristics of JrWRKY4 as a transcription activator located in the nucleus were first identified. Gain-of-function and loss-of-function analyses showed that JrWRKY4 could enhance walnut resistance against *C. gloeosporioides*. A series of molecular experiments showed that JrWRKY4 directly interacted with the promoter region of *JrSTH2L* and positively regulated its expression. In addition, JrWRKY4 interacted with JrVQ4 to form the protein complex, which inhibited JrWRKY4 for the activation of JrSTH2L. Notably, a MYB TF JrPHL8 interacting with the *JrWRKY4* promoter has also been identified, which directly bound to the MBS element in the promoter of *JrWRKY4* and induced its activity. Our study elucidated a novel mechanism of the JrPHL8-JrWRKY4-JrSTH2L in regulating walnut resistance to anthracnose. This mechanism improves our understanding of the molecular mechanism of WRKY TF mediated resistance to anthracnose in walnut, which provides new insights for molecular breeding of disease-resistant walnuts in the future.

## Introduction

The innate immune system of plants consists of two layers of immune mechanisms: pathogen-associated molecular pattern-triggered immunity (PTI) and effector-triggered immunity (ETI) [1–4]. The former is activated by plasma member-anchored pattern recognition receptors to detect conserved pathogen-associated molecular patterns (PAMPs, such as flagellin, EF-Tu, and chitin), which induced the accumulation of pathogenesis-related proteins (PRs) and the production of reactive oxygen species [5, 6]. In contrast, ETI is activated by NLRs that inhibit the growth and propagation of pathogens by inducing hypersensitive response cell death, thereby enhancing plant resistance [7, 8]. Although PTI and ETI differ in immune receptors, activation mechanisms, and early signaling components, they eventually converge in many similar downstream responses [9]. Recent research indicated that a strong defense response against pathogens can be achieved through signal collaboration between PTI and ETI mechanisms [9, 10]. In the process, transcription factors (TFs) adopt different immune activation and signal transduction mechanisms to participate in plant defense response [11].

WRKY TFs are the central component of the plant innate immune system, which participate in multiple signaling pathways through binding to the W-box in the target genes promoters in plants [12–15]. In recent years, the functions and molecular mechanisms of WRKY TFs in plant-pathogen interactions have been extensively studied. MdWRKY75 improved the resistance of apple roots to *Fusarium solani* by binding to the W-box motif in the *MdERF114* promoter [16]. VqWRKY31 confers resistance to *Erysiphe necator* in grapes by promoting salicylic acid signaling and the synthesis of specific metabolites [17]. VqWRKY56-VqbZIPC22 complex positively regulates grapevine resistance against *E. necator* by stimulating the synthesis of proanthocyanidins (PAs) [18]. Meanwhile, recent studies have shown that Valine-glutamine (VQ) proteins with the conserved VQ-motif structure FxxhVQxhTG can synergistically or antagonistically regulate various biotic and abiotic stress responses by interacting with WRKY proteins in plants [19, 20]. In Arabidopsis, AtVQ10 physically interacts with AtWRKY8 and enhances Arabidopsis resistance to *Botrytis cinerea* [21]. SlVQ15 and SlWRKY31 cooperatively regulate JA-mediated defense against *Botrytis cinerea* in tomato [22]. The interaction of OsVQ25 and OsWRKY53 suppresses OsWRKY53 transcriptional activity and OsWRKY53- downstream defense-related genes and brassinosteroid signaling genes to negatively regulate broad-spectrum resistance in rice [23].

Besides WRKY TFs, MYBs are also extensively involved in regulating plant growth and development, hormone signal transduction, secondary metabolism, biotic and abiotic stress through recognizing conserved MYB binding sites (MBSs) [24, 25]. AtMYB96 regulated salicylic acid (SA) biosynthesis and PR gene expression to enhance resistance to *Pseudomonas syringae* [26]. Arabidopsis knocking out AtMYB46 improves the mutants’ resistance to *B. cinerea* [27]. AtMYB44 as an integral component of the PTI pathway activated the expression of MPK3 and MPK6 by binding to their promoters and attributed to the activation of EIN2 transcription [28]. In pepper (*Capsicum annuum*), the MYB TFs CaPHL8 positively contribute to pepper’s resistance against *Ralstonia solanacearum* by upregulating the expression of PR genes [29]. VqMYB154 directly binds to the promoters of stilbene synthase genes VqSTS9, VqSTS32, and VqSTS42 to enhance grapevine resistance to *Uncinula necator* [30].

Walnut (*Juglans regia* L.) holds significant economic value as a valuable oil-producing tree species with kernels that are rich in fatty acid, protein, vitamins, and minerals [31–33]. However, walnut anthracnose caused by *Colletotrichum gloeosporioides* (Penz.) Penz. and Sacc. severely impacts both quantity and quality of walnuts, which has become the main factor restricting the healthy development of the walnut industry [34–36]. Recent advances in walnut functional genomics and molecular biology have provided a valuable technical platform to explore the mechanism of walnut resistance to *C. gloeosporioides*. Previous studies have shown that JrWRKY21 interacts with JrPTI5L and activates its activity by binding to the W-box element in the PTI5L promoter, while JrPTI5L activates the activity of JrPR5L by binding to the GCCGAC motif, which forms a cascade reaction promoting walnut resistance to *C. gloeosporioides* [37]. Meanwhile, the molecular mechanism of lncRNA109897 and its target gene JrCCR4 coregulating PR gene JrTLP1b expression to enhance walnut resistance to anthracnose has been elucidated [38]. Therefore, the molecular mechanism of TFs involved in walnut resistance to anthracnose deserves further investigation.

Our previous transcriptome analysis and qRT-PCR results found that *JrWRKY4* showed significantly higher expression in resistant walnut leaves L423 invaded by *C. gloeosporioides* than that in susceptible walnut leaves L37. Based on this, we further explored the regulatory pathway of JrWRKY4 in walnut resistance to anthracnose in this study. JrWRKY4 belongs to the group I subfamily and acts as a transcription activator located in the nucleus. The estimate of the disease resistance function of transgenic strains showed that JrWRKY4 enhances walnut resistance to anthracnose. A MYB TF JrPHL8 was identified through yeast one-hybrid screening, which binds to the MBS element on the JrWRKY4 promoter and promotes its transcription. Meanwhile, JrWRKY4 can bind to the W-box in the promoter of the downstream PR gene *JrSTH2L* and promote its expression. Additionally, the interaction between JrVQ4 and JrWRKY4 can inhibit the activation of JrWRKY4 on JrSTH2L. In summary, this study reveals the molecular mechanism of the JrPHL8-JrWRKY4- JrSTH2L module in regulating walnut resistance to anthracnose, further enriching the molecular understanding of walnut resistance to anthracnose and providing new insights for molecular breeding of disease-resistant walnuts.

## Results

### Expression pattern, phylogenetic analysis, and sequence alignment of JrWRKY4

**Figure 1:**
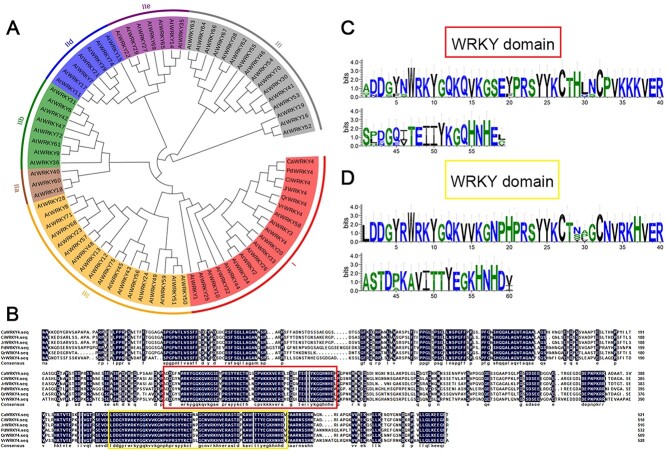
Expression pattern, phylogenetic analysis, and sequence alignment of *JrWRKY4*. **A** A phylogenetic tree constructed using the neighbor-joining method with 78 WRKY transcription factor (TF) proteins. **B** The amino acid sequences of six WRKY4 transcription factors, including the WRKY domain, were meticulously aligned for comprehensive comparison. **C** and **D** WRKY domains in JrWRKY4.

Our previous studies indicated that the expression pattern of *JrWRKY4* showed different trends in L423 (resistant walnut leaves) vs L37 (susceptible walnut leaves) during walnut resistance to *C. gloeosporioides*. qRT-PCR results showed that the transcription of *JrWRKY4* was at a higher level in L423 within 48 hpi (hours post inoculation) with *C. gloeosporioides* than that in L37 [37]. We then isolated it from L423 for future research. The phylogenetic tree was constructed with JrWRKY4, 72 *Arabidopsis thaliana* WRKY TFs, and WRKY4 TFs in other species via MEGA 5.1, which were clustered into three major groups. The results of phylogenetic tree analysis revealed that JrWRKY4 belongs to group I and has the highest homology with CiWRKY4 (LOC122307644) from *Carya illinoinensis* (Fig. 1a). The alignment of the amino acid sequences indicated that JrWRKY4 has a single 1554-bp open reading frame (ORF) and encodes a protein with 517 amino acids (Fig. 1b). As predicted, JrWRKY4 contained two WRKY domains (WRKYGQK), which were conserved at the C-terminus (Fig. 1c and d).

### Subcellular localization and transcriptional activation of JrWRKY4

**Figure 2:**
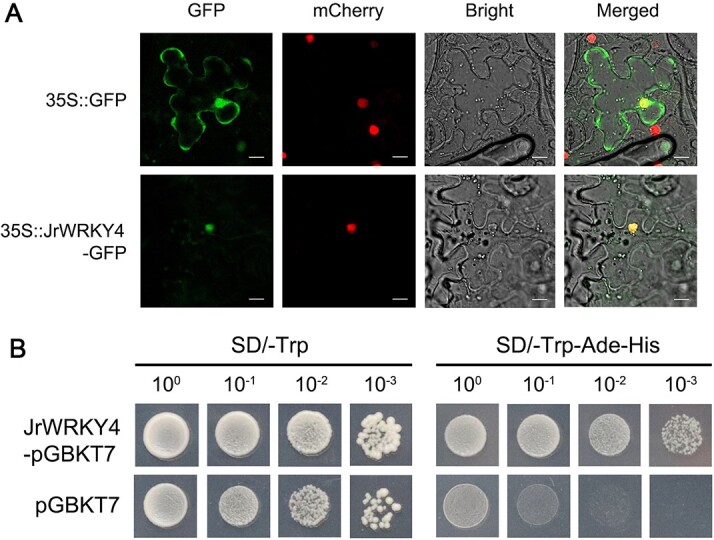
Subcellular localization and transcriptional activation of JrWRKY4. A Subcellular localization of JrWRKY4 protein in leaves of *Nicotiana benthamiana*. From left to right: green fluorescent protein (GFP), p2300–35 s-H2B-mcherry, bright-field, and merged. Bars = 2 μm. **B** Transcriptional activation of JrWRKY4 in yeast cells.

In order to investigate the subcellular localization of JrWRKY4, a fusion protein of JrWRKY4 and green fluorescent protein (GFP) was created. Then this fusion protein, along with the p2300–35 s-H2B-mcherry plasmid serving as a marker for nuclear localization, was co-transformed into the leaves of *Nicotiana benthamiana*. The JrWRKY4-GFP recombinant plasmid induced fluorescence only in the nuclei, whereas the fluorescent signals of the empty plasmid with GFP were observed in the cytoplasm and nucleus (Fig. 2a), indicating that JrWRKY4 was localized in the nucleus. Next, a yeast assay was performed to detect whether JrWRKY4 had transcription activation activity. The JrWRKY4-pGBKT7 recombinant plasmids and empty plasmid pGBKT7 (negative control) were transformed into Y2Hgold. Both JrWRKY4-pGBKT7 recombinant plasmids and empty plasmid transformed yeast grew well on the SD media lacking Trp (SD/−Trp), and only JrWRKY4-pGBKT7 recombinant plasmids grew well on SD media lacking Trp, His, and Ade (SD/−Trp-His-Ade), indicating that JrWRKY4 had the transcription activation (Fig. 2b). These results indicate that JrWRKY4 is a transcription activator located in the nucleus.

### JrWRKY4 enhances walnut resistance to *C. gloeosporioides*

**Figure 3:**
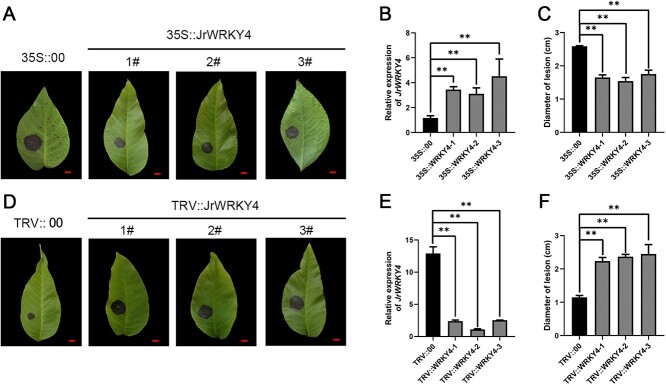
JrWRKY4 promotes walnut resistance to *Colletotrichum gloeosporioides.*  **A** Leaf phenotypes of 35S::JrWRKY4 walnut leaves infected with *C. gloeosporioides*. Bars = 1 cm. **B** Relative expression of JrWRKY4 in 35S::JrWRKY4 walnut leaves. **C** Diameter of lesions in 35S:: JrWRKY4 walnut leaves. **D** Leaf phenotypes of TRV::JrWRKY4 walnut leaves infected with *C. gloeosporioides*. Bars = 1 cm. **E** Relative expression of JrWRKY4 in TRV::JrWRKY4 walnut leaves. **F** Diameter of lesions in TRV:: JrWRKY4 walnut leaves. The 18S rRNA used as a housekeeping gene, ^*^P < 0.05, ^**^P < 0.01.

To further validate the anthracnose resistance function of JrWRKY4, the full CDS of JrWRKY4 was combined with pRI101 (negative control) to form JrWRKY4-pRI101 recombinant plasmids. We transformed JrWRKY4-pRI101 recombinant plasmids into ‘B37’ leaves by vacuuming and obtained transient overexpressed JrWRKY4 walnut (35S::JrWRKY4) (Fig. 3a), which has been proven to be successfully imported into plants by PCR amplification (Fig. S1, see online supplementary material). The qRT-PCR analysis revealed a significant increase of *JrWRKY4* gene in the leaves of 35S::JrWRKY4 walnut plants compared with controls (Fig. 3b). Then we used the detached leaf inoculation assay to detect the disease phenotypes of different strains. The 35S::JrWRKY4 walnut leaves that were inoculated with *C. gloeosporioides* displayed reduced disease symptoms and smaller lesions compared to the 35S::00 walnut leaves (Fig. 3a and c). In addition, we generated transgenic lines that overexpressed JrWRKY4 (OE-JrWRKY4) in the tobacco and tomato (Figs S2 and S3, see online supplementary material). The OE-JrWRKY4 transgenic lines were evaluated through PCR (Figs S2B and S3B, see online supplementary material) and qRT-PCR (Figs S2C and S3C). After *C. gloeosporioides* infection, the OE-JrWRKY4 transgenic line leaves displayed reduced disease symptoms than those of the WT, which suggested that JrWRKY4 could enhance the resistance to *C. gloeosporioides* (Figs S2A and S3A, see online supplementary material).

Meanwhile, the silent expression of JrWRKY4 walnut (TRV::JrWRKY4) were also obtained through virus-induced gene silencing (VIGS) technology. The expression of *JrWRKY4* in TRV::JrWRKY4 walnut leaves was significantly decreased compared with controls (Fig. 3e). PCR validation of TRV::JrWRKY4 was conducted using specific primer pairs pTRV1-F/R and pTRV2-F/JrWRKY4-R. In comparison to TRV::00-infected samples, the TRV::JrWRKY4-infected samples exhibited the amplification of a target band of 487 bp, indicating that the JrWRKY4-specific fragment was inserted (Fig. S2, see online supplementary material). Contrary to 35S::JrWRKY4 walnut inoculated with *C. gloeosporioides*, the TRV::JrWRKY4-transgenic walnut leaves displayed increased disease symptoms and larger lesions compared to the TRV::00-transgenic walnut leaves. (Fig. 3d and f). The above results indicate that JrWRKY4 can positively regulate walnut resistance to *C. gloeosporioides*.

### JrWRKY4 binds to the promoter of JrSTH2L and promotes its transcription

**Figure 4:**
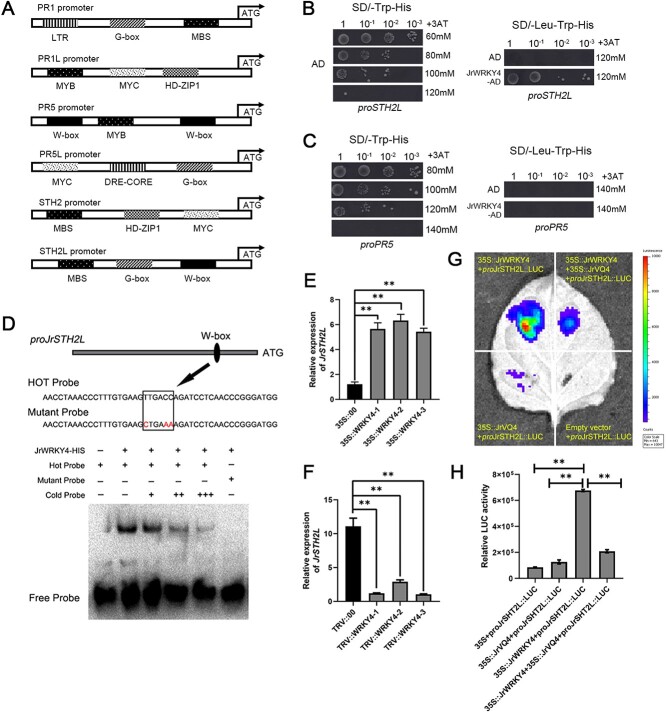
JrWRKY4 binds to the promoter of *JrSTH2L* and promotes its transcription. A Analysis of the promoter sequences of *JrPR1*, *JrPR1L*, *JrPR5*, *JrPR5L*, *JrSTH2*, and *JrSTH2L*. B and C Y1H assays show that JrWRKY4 binds to the promoter of *JrSTH2L* but not to the promoter of *JrPR5*. D EMSA indicates that JrWRKY4 binds to the W-box element (TTGACC) in the *JrSTH2L* promoter. Hot probe: biotin-labeled fragment containing W-box motif. Cold probe: non-labeled competitive probe (100-fold that of probe). The mutant probe contained three nucleotide mutations. E Relative expression of JrSTH2L in 35S::JrWRKY4 walnut leaves. F Relative expression of JrSTH2L in TRV::JrWRKY4 walnut leaves. G and H Dual-luciferase reporter assay shows that JrWRKY4 activates the promoter of *JrSTH2L*, but this activation is inhibited when JrWRKY4 interacts with JrVQ4.

The pathogenesis-related (PR) proteins have been reported to be essential in plant immune processes [39]. Our previous study has shown that JrPR1, JrPR1L, JrPR5, JrPR5L, JrSTH2, and JrSTH2L may be involved in walnut resistance to anthracnose [37]. To explore the relationship between JrWRKY4 and these PR proteins, we searched the PlantCARE database (http://bioinformatics.psb.ugent.be/webtools/plantcare/html/) and found that only *JrPR5* and *JrSTH2L* promoters contained W-box motif, which was putative WRKY-binding elements (Fig. 4a). To verify this hypothesis, a Y1H assay was conducted and yeast cells containing *pro*JrPR5-pHIS2 and *pro*JrSTH2L-pHIS2 were first grown on -Trp/-His screening medium to determine the optimal concentration of 3-amino-1,2,4-triazole (3-AT). The Y1H assay results showed that JrWRKY4 interacted with *JrSTH2L* promoter on the screening medium supplemented with -Leu/−Trp/-His and 120 mM 3AT, but JrWRKY4 did not interact with *JrPR5* promoter (Fig. 4b and c). Meanwhile, the EMSA results indicated that JrWRKY4 bound to the W-box element (TTGACC) in the *JrSTH2L* promoter (Fig. 4d).

Furthermore, we investigated the effect of JrWRKY4 on the transcriptional activity of JrSTH2L. Firstly, we found that the expression of *JrSTH2L* in 35S::JrWRKY4 walnut was also significantly increased, and correspondingly decreased in TRV::JrWRKY4 walnut (Fig. 4e and f). Next, we performed a dual-luciferase assay in tobacco (*N. benthamiana*) leaves. The CDS of JrWRKY4 was inserted into the pGreenII 62-SK vector as an effector. The promoter sequence of *JrSTH2L* was fused into the pGreenII 0800-LUC vector as a LUC reporter gene. LUC activity detection showed that the luminescence intensity of the coexpression of 35S::JrWRKY4 with *pro*JrSTH2L::LUC was six times higher than that of the individual expression of *pro*JrSTH2L::LUC (Fig. 4g and h). These results clearly indicated that JrWRKY4 can directly bind to the W-box of the *JrSTH2L* promoter and enhance its transcriptional activity.

### The interaction between JrWRKY4 and JrVQ4 inhibits JrSTH2L transcription

**Figure 5:**
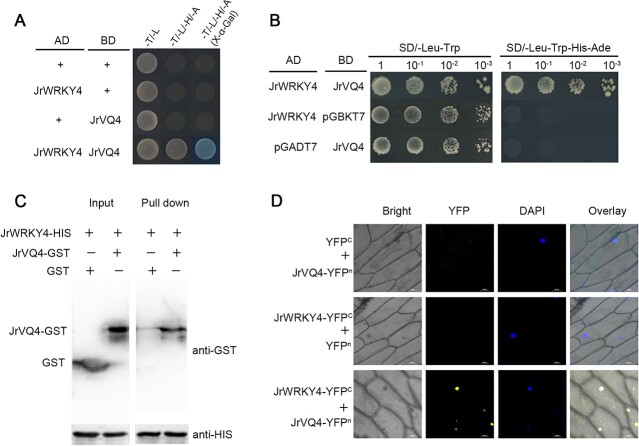
The interaction between JrWRKY4 and JrVQ4. **A** and **B** Y2H assay shows that JrWRKY4 interacts with JrVQ4. **C** Pull-down assay showing the interaction between JrWRKY4 and JrVQ4. The symbol ‘+’ indicates the presence of relevant proteins, while the symbol ‘-‘ indicates the absence of relevant proteins. **D** BiFC assay between JrWRKY4 and JrVQ4. The YFP field indicates fluorescence signals. Location of the nucleus is shown by staining with 4′,6-diamidino-2-phenylindole (DAPI).

To gain deeper insights into the involvement of JrWRKY4 in walnut resistance against *C. gloeosporioides*, a yeast two-hybrid (Y2H) screening assay was conducted to determine interacting proteins from the cDNA library of walnut using the JrWRKY4 protein as a bait. JrVQ4 (LOC109013141) was filtered as a potential JrWRKY4 interacting protein. To confirm the interaction between JrWRKY4 and JrVQ4, Y2H, Pull-down, and biomolecular fluorescence complementation (BiFC) assays were conducted. The results from Y2H assay demonstrated that JrWRKY4 and JrVQ4 interacted with each other (Fig. 5a and b). After copurification of the JrWRKY4-HIS fusion protein and JrVQ4-GST fusion protein, a pull-down assay was conducted. JrWRKY4-HIS was observed to be captured by JrVQ4-GST, indicating that JrWRKY4 interacts with JrVQ4 (Fig. 5c). The BiFC assay was conducted to confirm that JrWRKY4 interacted with Jr VQ4 *in vivo* (Fig. 5d).

Increasing evidence indicates that WRKY-VQ complexes synergistically or antagonistically regulated plant growth and stress resistance [40, 41]. Therefore, we examined whether the JrVQ4 has any effects on the JrWRKY4 activation of *JrSTH2L*. The CDS of JrVQ4 was also inserted into the pGreenII 62-SK vector as an effector. As shown in Fig. 4g and h, LUC assay results showed that 35S::JrWRKY4 activation of the *pro*JrSTH2L::LUC activity was almost fully suppressed when 35S::JrWRKY4, 35S::JrVQ4 and *pro*JrSTH2L::LUC coinfected tobacco leaves(Fig. 4g and h). These data manifest that JrVQ4 represses the JrWRKY4 activation of *JrSTH2L* by interacting with JrWRKY4.

### JrPHL8 binds to JrWRKY4 promoter and enhances its transcription

**Figure 6:**
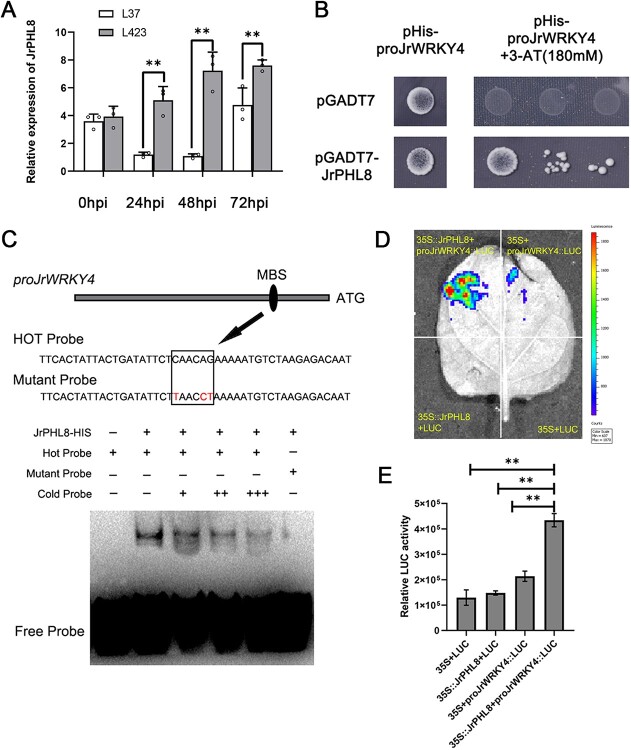
JrPHL8 binds to *JrWRKY4* promoter and enhances its transcription. **A** Relative expression of JrPHL8 in L37 and L423 plants inoculated with *Colletotrichum gloeosporioides* within 0–72 hpi. **B** Y1H assay revealed the binding of JrPHL8 to the promoter region of *JrWRKY4*. **C** EMSA indicates that JrPHL8 binds to the MBS elements (CAACAG) in the *JrWRKY4* promoter. Hot probe: biotin-labeled fragment containing W-box motif. Cold probe: non-labeled competitive probe (100-fold that of probe). The mutant probe contained three nucleotide mutations. **D** and **E** Dual-luciferase reporter assay shows that JrPHL8 activates the promoter of *JrWRKY4*.

To further investigate the upstream regulatory factors of JrWRKY4, a yeast one-hybrid (Y1H) screening assay was conducted using the *JrWRKY4* promoter. Fortunately, the MYB transcription factor JrPHL8 (XM_018951889.2) was identified as binding to the WRKY4 promoter. To further investigate whether the expression of JrPHL8 is directly induced by *C. gloeosporioides*, we used RT-qPCR to detect the expression levels of JrPHL8 in the leaves of walnut susceptible variety L37 and resistant variety L423 at different infection stages (0 hpi, 24 hpi, 48 hpi, 72 hpi). The results showed that the expression levels of JrPHL8 were higher in L423 leaves than that in L37 at 24 hpi, 48 hpi, and 72 hpi (Fig. 6a). This indicates that *C. gloeosporioides* can directly induce the expression of JrPHL8.

To confirm the binding relationship between PHL8 and WRKY4 promoter, we cloned the CDS of JrPHL8 and promoter fragments of *JrWRKY4* into the pGADT7 and pHIS2 vectors respectively to perform a Y1H assay. The yeast cells co-transformed with *pro*JrWRKY4-pHIS2 and JrPHL8-pGADT7 could grow on the SD/−Leu-Trp-His medium containing 180 mM 3AT, but the negative control co-transformed with *pro*JrWRKY4-pHIS2 and pGADT7 did not grow (Fig. 6b), indicating that JrPHL8 interacts with *JrWRKY4* promoter. Next, we utilized the PlantCARE database to analyze the *cis*-acting elements in the *JrWRKY4* promoter and found a putative MYB-binding element (MBS) (Fig. S3, see online supplementary material). The EMSA assay using purified recombinant JrPHL8-HIS and the probe of MBS was performed. The results indicated that JrPHL8 directly bound to the MBS elements (CAACAG) of the *JrWRKY4* promoter (Fig. 6c). Furthermore, the effect of JrPHL8 on the promoter activity of *JrWRKY4* was also validated in tobacco leaves using a dual-luciferase assay. The results showed that the coexpression of 35S::JrPHL8 with *pro*JrWRKY4::LUC emerged with significantly stronger luminescence intensity compared with the negative controls and the individual expression of *pro*JrWRKY4::LUC (Fig. 6d). These results determined that JrPHL8 can directly bind to the MBS on the *JrWRKY4* promoter and significantly enhance the transcription of JrWRKY4.

## Discussion

Anthracnose in walnuts can lead to a significant reduction in yield, causing up to a 50% loss, and can have a detrimental impact on the walnut industry [34, 42]. Due to environmental and cost issues caused by chemical control, molecular breeding based on elucidating the molecular mechanism of disease resistance has become the most effective strategy for disease control. Recent extensive study into walnut omics has provided convenience for identifying resistance genes and TFs related to walnut anthracnose resistance. Many chorismate and phenylalanine pathways-related genes *PBS3*, *S5H*, *C4H*, and *COMT* were identified through transcriptome analyses during *C. gloeosporioides* infected walnut fruits [43]. *JrMAPK* genes were also found to be up up-regulated expression in response to different levels of *C. gloeosporioides* infection [44]. WRKY TFs as an important component of plant immunity have also been identified in the walnut–*C. gloeosporioides* interaction. Compared with anthracnose-susceptible F423 fruits, *JrWRKY83, JrWRKY73,* and *JrWRKY74* showed significant upregulation of expression in anthracnose-resistant F26 fruits [45]. However, the specific function and molecular mechanism of WRKY TFs in walnut resistance to anthracnose was poorly understood. Herein, we focused on the characteristic and function of JrWRKY4 based on previous transcriptome analysis and qRT-PCR results. Subcellular localization and transcriptional activation assays indicated JrWRKY4 was a transcription activator located in the nucleus. Evaluation of disease resistance in 35S::WRKY4 and TRV::WRKY4 transgenic strains showed that JrWRKY4 as a positive regulatory factor enhances walnut resistance against *C. gloeosporioides*. Therefore, the molecular mechanism of JrWRKY4 underlying walnut anthracnose resistance needs to be given an in-depth investigation.

WRKY TFs regulate their transcriptional activity by binding to W-box element (T)TGAC(C/T) of downstream disease-related target genes [46, 47]. Group IIc WRKY TFs directly bound to the W-box in the promoter of *GhMKK2* and promoted GhMKK2-mediated flavonoid biosynthesis to regulate cotton resistance to *Fusarium oxysporum* [48]. Among the downstream target genes regulated by WRKY TFs, the PR gene as a marker gene associated with plant-pathogen responses is widely identified. Our previous study indicated that six PR genes (*JrPR1*, *JrPR1L*, *JrPR5*, *JrPR5L*, *JrSTH2*, and *JrSTH2L*) related to walnut resistance to anthracnose [37]. In this study, we found that only *JrPR5* and *JrSTH2L* promoters contained W-box motifs. A series of yeast one-hybrid assay electrophoresis mobility shift assay and dual-luciferase assay experiments showed JrWRKY4 could promote the transcriptional activity of *JrSTH2L* by interacting with the W-box (TTGACC) in promoter. Similar to the previous findings on SlWRKY30 and SlWRKY81, it has been observed that these two transcription factors work synergistically to regulate tomato resistance against bacterial wilt caused by *R. solanacearum*, and one of the mechanisms by which they exert this regulation is through the activation of SlPR-STH2a/b/c/d expression [49].

Meanwhile, WRKY TFs often form complexes with VQ proteins to activate or de-repress the transcription of target genes. The latest research indicated that SlVQ16 interacted with SlWRKY75 and enhanced SlWRKY75-mediated transcriptional activation of *SlGH3.3*, therefore promoting tomato defense against *Pst* DC3000 [50]. The formation of the BnaA03.WRKY28-BnaA09.VQ12 protein complex led to the binding of BnaA03.WRKY28 to the promoter region of *BnWRKY33*, decreased BnWRKY33 expression to suppress the oilseed rape resistance to *Brassica napus* [51]. OsVQ25 interacts with OsWRKY53 to suppress OsWRKY53 transcriptional activity for downstream defense-related genes and brassinosteroid signaling genes, thereby negatively regulating broad-spectrum resistance in rice [23]. In this study, we identified the JrWRKY4-interacting protein JrVQ4 through the yeast two-hybrid (Y2H) screening assay, which was verified by Pull-Down and BiFC experiments. LUC assay results indicated that JrVQ4 represses the JrWRKY4 activation for *JrSTH2L* by the interaction. Our results as well as previous research have indicated that the WRKY-VQ protein complex was vital to plant resistance against pathogens.

Although the interaction between WRKY transcription factors and other TFs in plant immunity has been extensively studied, research on regulating WRKY TF activity is mostly limited to the MAPK signaling pathway [52, 53]. Ichimaru *et al.* found that the transcriptional activation of WRKY45 is mediated by the synergism between MAP kinase-regulated phosphorylation and PUB44-regulated PBI1 degradation in rice immunity [54]. Additionally, the clock protein GmTOC1b binds to the promoter of *GmWRKY40* and inhibits *GmWRKY40* expression, which reduces Soybean (*Glycine max*) resistance to *Soybean mosaic virus* (SMV) [55]. In our study, we found that a MYB TF JrPHL8 can bind to the MBS elements (CAACAG) of the *JrWRKY4* promoter and activate the transcriptional activity of *JrWRKY4*. Numerous previous studies have confirmed that MYB TFs also play an important role in plant immunity. GmMYB29A2 directly binds to the MBS elements in the promoters of two glyceollin I biosynthesis genes *G4DT* and *IFS2* to regulate soybean resistance to *Phytophthora sojae* [56]. The Myb-like TF REVEILLE2 (RVE2) regulated the jasmonic acid (JA) pathway to confer cotton resistance against *Verticillium wilt* (VW) [57].

**Figure 7:**
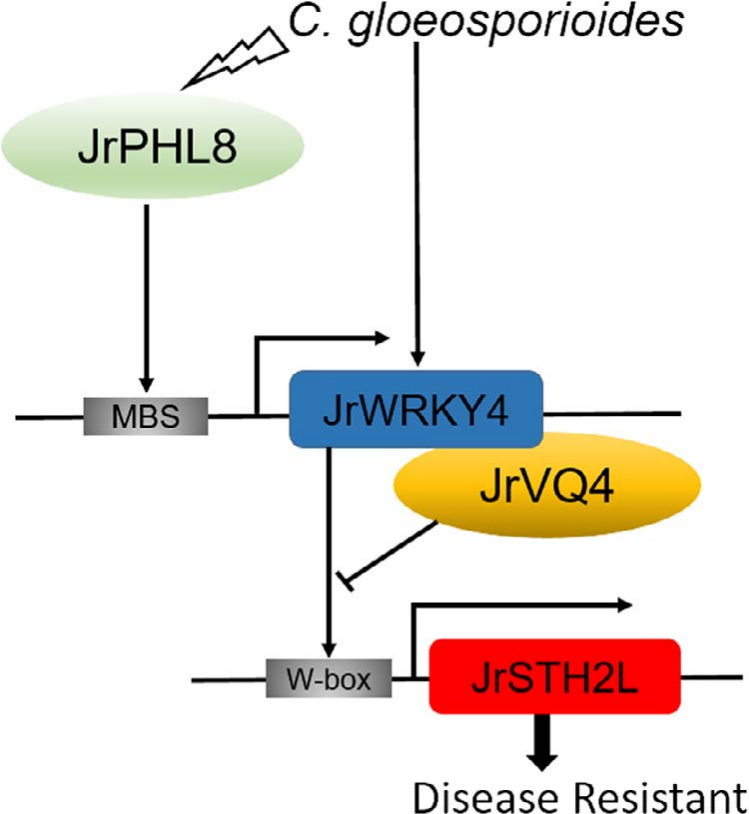
A working model illustrating the role of JrPHL8-JrWRKY4-JrSTH2L in walnut response to *Colletotrichum gloeosporioides*.

Taken together, our research findings elucidate a novel mechanism in which the JrPHL8-JrWRKY4-JrSTH2L pathway regulates walnut defense against *C. gloeosporioides* infection. During the infection process of *C. gloeosporioides*, JrWRKY4 enhances the transcriptional activity by binding to the W-box on the *JrSTH2L* promoter, thereby positively regulating the resistance of walnut to *C. gloeosporioides*. In addition, the interaction between JrWRKY4 and JrVQ4 inhibits the activation of *JrSTH2L* by JrWRKY4. Furthermore, in the course of *C. gloeosporioides* infection, JrPHL8 is induced and enhances its transcriptional activity by binding to the MBS element on the *JrWRKY4* promoter, thereby positively regulating walnut resistance to *C. gloeosporioides* (Fig. 7). This disease resistance mechanism further enhances our understanding of the regulatory network mediated by WRKY TFs in walnut resistance to anthracnose.

## Materials and methods

### Plant materials and growth conditions

The plant materials used for VIGS and transient overexpression in this study were the anthracnose-disease walnut clone ‘4–23’ and the susceptible walnut clone ‘B37’, which have been depicted in our previous research [37]. ‘4–23’ are served as VIGS control (TRV:00) and B37 (35S:00) served as transient overexpression control.

Tobacco (*N. benthamiana*) and Tomato (*Solanum lycopersicum* ‘Micro Tom’) seedlings were cultivated in soil in a growth chamber with a 16 h light/8 h dark photoperiod with light intensity 120 μmol m^−2^ s^−1^ at 25°C. The strain ‘m9’ of *C. gloeosporioides* (GenBank ID: GU597322) used in this study was maintained by our laboratory [58].

### Bioinformatic analysis

We used DNAMAN and SMART software to conduct the analysis of the JrWRKY4 protein sequence and simultaneously constructed a phylogenetic tree using MEGA.

### Subcellular localization

To obtain a recombinant vector, a GFP tag was inserted into the CDS of JrWRKY4. The GFP-JrWRKY4, empty GFP, and p2300–35 s-H2B-mcherry constructs were introduced into *Agrobacterium* GV3101-Psoup-P19 through transformation. After shaking the cultures until OD_600_ = 0.8, the GFP-JrWRKY4 and empty GFP *Agrobacterium* cultures were mixed in a 1:1 ratio with the p2300–35 s-H2B-mcherry *Agrobacterium* culture. The mixtures were then separately infiltrated into 4-week-old tobacco leaves and incubated in the dark for 48 hours. The green fluorescence signal was visualized using LSM880 (Carl Zeiss, Oberkochen, Germany). The primers used for the amplification are listed in Table S1 (see online supplementary material).

### Transcriptional activation assay in yeast

The CDS of JrWRKY4 was cloned into the pGBKT7 vector. The resulting construct, pGBKT7-JrWRKY4, was transformed into the yeast strain Y2H Gold (Weidi Biotechnology, Shanghai, China). The pGBKT7 vector was used as a negative control. The transformed yeast cells were plated on SD/−Trp medium. Colonies showing successful transformation were subsequently transferred to selective SD/−Trp/-His/ -Ade medium supplemented with X-α-Gal and incubated at 28°C for three days to assess transcriptional activity. The primers used for the amplification are listed in Table S1 (see online supplementary material).

### VIGS in walnut leaf tissues and analysis of disease symptoms

Specific fragments of JrWRKY4 were amplified from the leaf cDNA of ‘4–23’ walnut, and the clone product from pTRV2-JrWRKY4 was inserted into the pYL156 vector. The pTRV1, pTRV2, and pTRV2-JrWRKY4 were transferred into *Agrobacterium* GV3101. Then using a mixed culture of *Agrobacterium* to infect the young leaves of ‘L-423’ walnut with VIGS, following the methods described in Zhou *et al.* [38]. Each treatment was performed with three biological replicates, and within each replicate, three leaves were measured. The primers used for the amplification are listed in Table S1 (see online supplementary material).

### Gene transient overexpression and analysis of disease symptoms

The CDS of JrWRKY4 was inserted into the PRI-101 vector to construct the recombinant expression vector. Then it was transfered along with the empty PRI-101 vector into *Agrobacterium* GV3101. The *Agrobacterium* carrying 35S::00 and 35S::JrWRKY4 genes were introduced into the young leaves of ‘B-37’ walnut, tobacco, and tomato, respectively, following the methods described in Sun *et al.* [11], then conducted disease resistance analysis as described by VIGS. Each treatment was performed with three biological replicates, and within each replicate, three leaves were measured. The primers used for the amplification are listed in Table S1 (see online supplementary material).

### RNA extraction and qRT-PCR

The specific methodology followed the approach described in Zhou *et al.* [38]. The primers for RT-qPCR were synthesized by Sangon Biotech (Shanghai, China; Table S2, see online supplementary material; http://www.sangon.com).

### Yeast one-hybrid screening library in walnut leaves

DNA was isolated from ‘4–23’ walnut trees, the specific primer was designed to clone the promoter region of JrWRKY4 for constructing a bait vector (pAbAi). The primers used for the amplification are listed in Table S1 (see online supplementary material). The linearized plasmid was introduced into yeast Y1H competent cells, then the cDNA library screening was conducted following the instructions provided in the user manual (Oebiotech, Shanghai, China).

### Yeast one-hybrid assays

JrWRKY4 and JrPHL8 were inserted into the pGADT7 vector, and the promoter fragments of JrPR5, JrSTH2L, and JrWRKY4 were cloned and ligated into the pHIS2 plasmid, respectively. The optimal concentration of 3-AT for each recombinant vector was screened on SD/−Trp/-His plates. Subsequently, they were co-transformed onto SD/−Trp/-His/−Leu plates to assess their interactions. The primers used for the amplification are listed in Table S1 (see online supplementary material).

### Electrophoretic mobility shift assays (EMSA)

The CDSs of JrWRKY4 and JrPHL8 were cloned and introduced into pET32a. The primers used for the amplification are listed in Table S1 (see online supplementary material). The recombinant plasmids were introduced into *Escherichia coli* BL21Condon plus (DE3) cells for protein expression and subsequent purification. We used the LightShift Chemiluminescent EMSA Kit (Beyotime) to perform EMSAs. The probe sequences, labeled with biotin, are listed in Table S3 (see online supplementary material). Mutant probes, containing three nucleotide mutations, were also labeled for specific experiments.

### Dual-luciferase assays

The CDSs of JrWRKY4, JrVQ4, and JrPHL8 were inserted into the pGreenII 62-SK plasmid as effectors, the promoter sequences of JrWRKY4 and JrSTH2L were recombined into the pGreenII 0800-LUC plasmid as reporters. The above recombinant plasmids were transformed into *Agrobacterium* GV3101-Psoup-P19, resuspend in infiltration buffer to OD_600_ = 1.0, then mixed in equal proportions, infecting the four-week-old tobacco leaves. The infiltrated leaves of *N. benthamiana* were cultured in the dark for 2 d and then sprayed with 500 μM luciferin and placed in the dark for 5 min. A living imaging analysis system (NightOWL II LB983; Berthold) was used for LUC activity determination. The primers used for the amplification are listed in Table S1 (see online supplementary material).

### Yeast two-hybrid screening library in walnut leaves

The CDS of JrWRKY4 was inserted into the pGADT7 vector. The primers used for the amplification are listed in Table S1 (see online supplementary material). Specific methods can be found in Zhou *et al.* [38].

### Yeast two-hybrid (Y2H) assay

To create the recombinant vector, we connected the CDS of JrWRKY4 to pGADT7, and the CDS of JrVQ4 to pGBKT7, then co-transformed them to Y2H yeast cells (Weidi Biotechnology, Shanghai, China) competent cells and developed on SD/−Trp/−Leu and SD/−Trp/−Leu-His/−Ade medium for 5 days, then the yeast colonies transformed with the recombinant vectors were plated on selective medium (SD/-His/−Ade/−Trp/−Leu) supplemented with X-α-Gal. The plates were then incubated for 5 days to allow the growth of new plaques, indicating potential protein–protein interactions. The primers used for the amplification are listed in Table S1 (see online supplementary material).

### Pull-down assay

The CDS of JrWRKY4 was cloned into pET32a, which contains the His-tagged sequence. The CDS of JrVQ4 was inserted into pGEX-4 T-1 with a GST-tagged sequence. The recombinant vector plasmids were transferred into *E. coli* BL21Condon plus (DE3) for protein expression and then purified. Next, they were verified by Western blotting. The primers used for the amplification are listed in Table S1 (see online supplementary material).

### Bimolecular fluorescence complementation (BIFC) assay

In order to construct the recombinant vector, we connected the CDS of JrWRKY4 to 35S-pspyCe-YFP, and the CDS of JrVQ4 to 35S-pspyNe-YFP. The resulting recombinant vectors were then transformed into *Agrobacterium* GV3101 (Weidi Biotechnology, Shanghai, China), which were also co-injected into onion epidermal cells for 24–48 h. The Zeiss LSM 880 laser scanning confocal microscope was used to observe fluorescent signals. The primers used for the amplification are listed in Table S1 (see online supplementary material).

## Supplementary Material

Web_Material_uhae148
