# Supplementary material for: JrPHL8-JrWRKY4-JrSTH2L module regulates resistance to Colletotrichum gloeosporioides in walnut
Source: Hortic Res. 2024 May 28;11(7):uhae148. doi: 10.1093/hr/uhae148 (PMC11233879; doi:10.1093/hr/uhae148)
Supplement: Web_Material_uhae148 [file web_material_uhae148.zip › supplymental figure.docx]

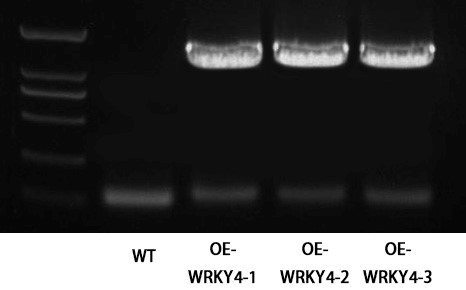


Figure S1. PCR validation of overexpression of JrWRKY4 in walnut.


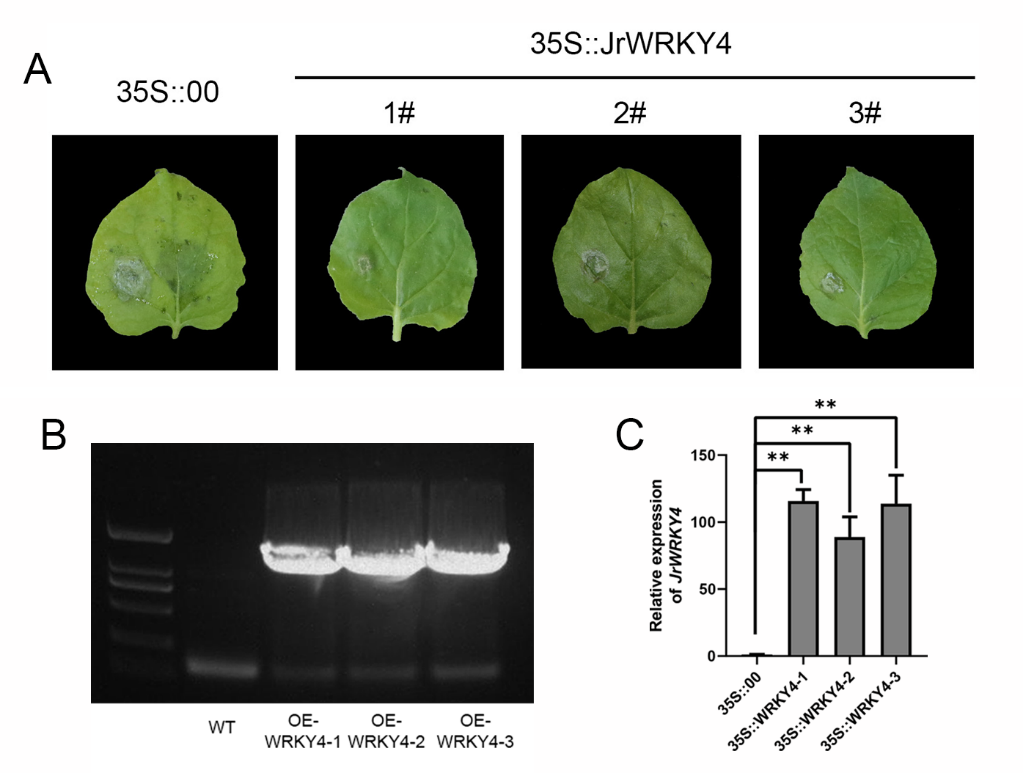


Figure S2. Heterologous overexpression of JrWRKY4 enhances disease resistance in tobacco leaves.

A. Symptoms of tobacco leaves overexpressing JrWRKY4 after inoculation, B. PCR validation of over expression of JrWRKY4 in tobacco leaves, C. qRT-PCR validation of over expression of JrWRKY4 in tobacco leaves.


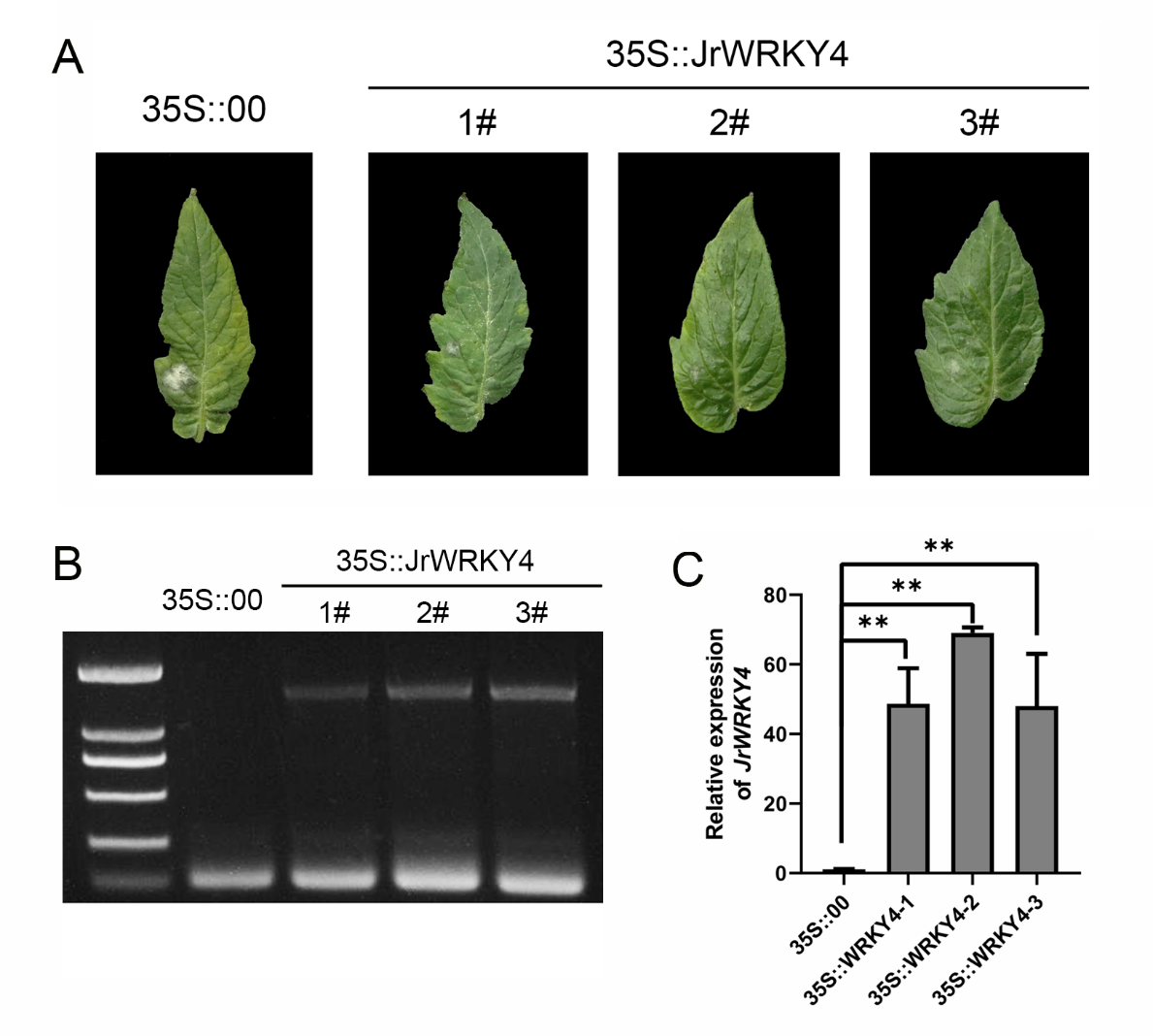


Figure S3. Heterologous overexpression of JrWRKY4 enhances disease resistance in tomato leaves.

A. Symptoms of tomato leaves overexpressing JrWRKY4 after inoculation, B. PCR validation of over expression of JrWRKY4 in tomato leaves, C. qRT-PCR validation of over expression of JrWRKY4 in tomato leaves.


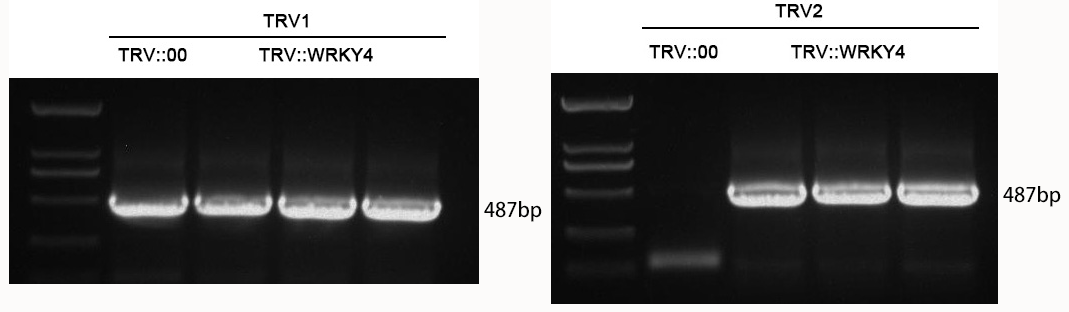


Figure S4. PCR validation of silent expression of JrWRKY4 in walnut.


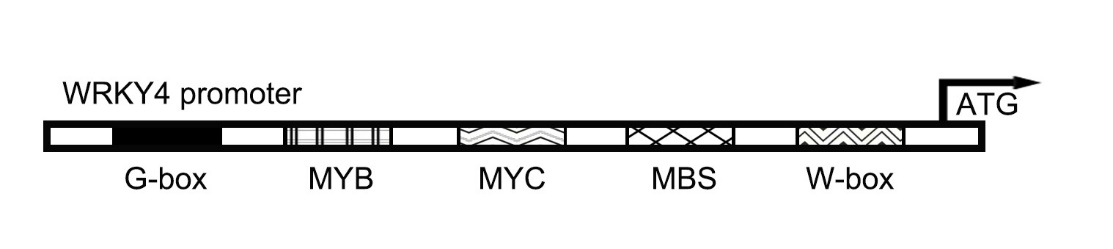


Figure S5. Schematic diagram of cis-regulatory elements in the JrWRKY4 promoter.
